# Dynamic relationship among extracellular matrix and body wall cells in *Hirudo verbana* morphogenesis

**DOI:** 10.1007/s00441-024-03874-x

**Published:** 2024-03-01

**Authors:** Laura Pulze, Nicolò Baranzini, Francesco Acquati, Gaia Marcolli, Annalisa Grimaldi

**Affiliations:** 1 Department of Biotechnology and Life Sciences, University of Insubria, via J.H. Dunant 3, 21100 Varese, Italy; 2 ILFARM s.r.l., via Guicciardini 14, 21100 Varese, Italy

**Keywords:** Microenvironment, Extracellular matrix, Telocytes, Leech, Development, Collagen, Muscle cells, RNASET2

## Abstract

A great bulk of recent experimental evidence suggests the key role of the complex crosstalk between the extracellular matrix (ECM) and the cellular component of tissues during morphogenesis and embryogenesis. In particular, remodeling of the ECM and of its physical interactions pattern with surrounding cells represent two crucial processes that might be involved in muscle development. However, little information is available on this topic, especially on invertebrate species. To obtain new insights on how tuning the ECM microenvironment might drive cellular fate during embryonic development, we used the invertebrate medicinal leech *Hirudo verbana* as a valuable experimental model, due to its simple anatomy and the recapitulation of many aspects of the basic biological processes of vertebrates. Our previous studies on leech post-embryonic development have already shown the pivotal role of ECM changes during the growth of the body wall and the role of Yes-associated protein 1 (YAP1) in mechanotransduction. Here, we suggest that the interactions between stromal cell telocytes and ECM might be crucial in driving the organization of muscle layers during embryogenesis. Furthermore, we propose a possible role of the pleiotropic enzyme *Hv*RNASET2 as a possible modulator of collagen deposition and ECM remodeling not only during regenerative processes (as previously demonstrated) but also in embryogenesis.

**Supplementary Information:**

The online version contains supplementary material available at 10.1007/s00441-024-03874-x.

## Introduction

In recent years, a growing interest in developmental biology has converged on investigating how the cellular and molecular crosstalk between cells and the surrounding microenvironment modulates the different stages of embryonic and post-embryonic development. To date, most published data concern commonly used vertebrate animal models, with very few studies focused on invertebrate species (Walma and Yamada 2020).

However, there are several compelling reasons for which invertebrate species, such as *Hirudo verbana* (Annelida, Hirudinea), should be considered as valuable experimental models for investigations on embryonic development. First, *H. verbana* displays analogous cell-cell and cell-matrix interactions (de Eguileor et al. 1999) and uses molecular effectors similar to those of vertebrates, at both cellular and biochemical levels (Pulze et al. 2022). Furthermore, investigations on leech development are important to provide experimentally accessible examples of developmental phenomena that are otherwise difficult to elucidate in more complex model systems. Indeed, in the leech body wall, there are few cell types, already well characterized in the adult (see supplementary fig. 2), whose differentiation is easily to follow during embryonic development. In addition, studying the embryonic development of leeches could help shed light on the crosstalk mechanisms that occur between embryonic cells and different components of the extracellular matrix (ECM).

Our previous studies on leech post-embryonic development (from hatching up to the adult stage) have already reported interesting data on this topic (Pulze et al. 2022). In particular, we showed that newborn leeches, though very small, are able to swim and crawl even if they widely differ from completely developed animals in terms of body wall thickness, number of muscle cells, degree of differentiation, and, most interesting, stiffness of the ECM. Despite these differences, the overall structural organization of the body and the spatial disposition of myocytes can be easily discerned in newborn leeches just after hatching.

Here, we extended our previous investigations on leech development further back in time, in order to examine key developmental processes taking place within the leech cocoons, produced after mating and containing about 15–18 mobile embryos.

In particular, we were interested in reporting and elucidating the occurrence of physical interactions between stromal cells [in particular telocytes (TCs)] and the ECM, which might be involved in the organization of muscle layers during embryogenesis, for instance, by guiding precursor muscle cells in the formation of the correct three-dimensional organization of muscle tissue.

To date, TCs have been described in many tissues/organs in both invertebrates (Pulze et al. 2017) and vertebrates (including humans) (Popescu and Faussone-Pellegrini 2010; Cretoiu et al. 2013, 2015; Albulescu et al. 2015; Aleksandrovych et al. 2017; Kondo and Kaestner 2019). TCs, generally localized in interstitial spaces, are characterized by a small cell body containing organelles and nucleus, with very thin and long prolongations, called telopodes (TPs). These cells establish homocellular contacts with other TCs but also heterocellular contacts with different cell types, forming a 3D network embedded in the ECM (Pulze et al. 2017; Hussein and Mokhtar 2018). Of note, the crosstalk among TCs and neighboring cells, coupled to the reciprocal exchange of several signaling molecules (Smythies and Edelstein 2014), has been reported to influence many key biological processes. These cell-to-cell contacts can take place both directly (*via* gap junctions) and indirectly, *via* extracellular vesicles such as multivesicular bodies and exosomes delivering different molecules (Aleksandrovych et al. 2017; Pulze et al. 2017).

Given their functional features, several roles have been attributed to TCs, including the regulation of immune response (Wollheim 2016), the control of key developmental steps within many tissues, and the modulation of the differentiation program during vertebrate embryogenesis (Fertig et al. 2014; Soliman 2017) (by means of driving stem cell migration and modulating their response to external cues) (Shoshkes-Carmel et al. 2018). Indeed, a role of TCs in organs and tissue development has already been reported in the developing rabbit’s lung tissue, where the number of TCs significantly increases to form an extensive network of telopodes (Hussein and Mokhtar 2018). Furthermore, other studies suggest that TCs might regulate the differentiation program of cardiomyocytes and myogenic precursors during vertebrate embryogenesis (Fertig et al. 2014; Soliman 2017).

As far as the ECM is concerned, it has long been established that it represents a complex, multifunctional component of most tissues, endowed with the ability to influence both biochemical and morphogenetic events such as cell growth, differentiation, and survival (Rozario and DeSimone 2010). The ECM is composed by a complex assembly of different families of dynamically interconnected molecules (Gordon and Hahn 2010; Pompili et al. 2021). It is able to modulate cellular responses to external stimuli as well as to regulate intracellular responses involved in cell homeostasis (Frantz et al. 2010; Theocharis et al. 2016; Pompili et al. 2021). In particular, the ECM plays a crucial role during tissue development by influencing crucial cell behaviors such as cell adhesion, proliferation, survival, migration, polarity, and differentiation in both an invertebrate model (*H. verbana*) (Pulze et al. 2022) and vertebrates (Pompili et al. 2021).

Noteworthy, during embryonic development different classes of ECM molecules, such as collagens, adhesion molecules, proteoglycans, and integrins, interact with neighbor cells to promote or restrict their migration, limit the diffusion of morphogens, and define cell boundaries. Furthermore, the physical properties of the ECM, such as its topography, composition, and stiffness, are sensed by surrounding cells and converted into cellular responses in the process of mechanotransduction (Jansen et al. 2015, 2017; Doyle and Yamada 2016; Ringer et al. 2017; Chighizola et al. 2019; Pulze et al. 2022). In this frame, a growing interest has been focused in recent years on elucidating the cellular and molecular mechanisms underlying mechanotransduction (i.e., the ability of a cell to transduce mechanical signals) (Jansen et al. 2015, 2017; Doyle and Yamada 2016; Ringer et al. 2017; Chighizola et al. 2019; Walma and Yamada 2020) and has unveiled the involvement of the Hippo pathway effectors YAP1 (Yes-associated protein 1) and TAZ (transcriptional coactivator with PDZ binding motif) in converting mechanical signals into specific transcriptional outputs (Cai et al. 2021; Pulze et al. 2022). Indeed, changes in ECM stiffness or loss of cell contact and stretching can induce the translocation of YAP/TAZ into the nucleus, followed by the activation of a specific transcriptional program, as we demonstrated also in hatched leech development (Pulze et al. 2022).

Despite the growing evidence pointing at TCs as key cellular effectors to modulate embryonic tissue development and differentiation, the detailed characterization of the precise contribution of TCs on ECM biodynamic and mechanotransduction still represents a critically unmet need.

For this purpose, *H. verbana* represents a useful and valuable experimental model as its use in experimental biology poses minimal ethical concerns. Furthermore, the embryonic developmental stages are easy to stage and manipulate, and their morphology has already been studied, well characterized, and described (Kutschera and Elliott 2014). Another interesting aspect is that TCs have been identified (Pulze et al. 2017) in the leech ECM, whose composition has been previously well characterized (de Eguileor et al. 1999; Tettamanti et al. 2005; Pulze et al. 2022). In addition, our previous studies on the postembryonic development of the leech (from hatching to adult) have also demonstrated the key role played by the ECM and its change in stiffness during the development and growth of the body wall. Finally, two key molecules involved in leech ECM remodeling have been identified in leeches: *Hv*RNASET2 (Baranzini et al. 2019) regulates the expression of both pro-collagen 1α1 (COL1α1) and basic fibroblast growth factor receptors (bFGFR, a known marker of fibroblasts) and YAP1, a key mediator converting ECM mechanical signals into transcriptional outputs, allows cells to perceive and respond to ECM changes (Pulze at al. 2022).

Taken together, these data prompted us to further extend our investigations on the occurrence of a crosstalk between the ECM and embryonic cells in a still unexplored phase of *H. verbana* development.

## Material and methods

All experiments were performed in three independent replicates. Unless otherwise indicated, all chemicals were purchased from Sigma–Aldrich (Saint Louis, MO, USA).

### Leech maintenance and dissections

The cocoons, containing each 15–18 embryos of leeches (*Hirudo verbana*, Annelida, Hirudinea), were bought from Ricarimpex (Eysines, France) and housed on humid moss in aerated tanks, in an incubator at a controlled temperature of 20 °C.

The cocoons are structures formed by the clitellum, in which the fertilized eggs are passed from the female pore, and they are then laid down by the leech. At different time points from deposition, the cocoons were dissected under a stereo light microscope and embryos were staged according to Reynolds and co-workers (Reynolds et al. 1998): the age of the embryos is defined as a percent of the time between cocoon deposition and the appearance of the ventrolateral stripe (100%; the latest significant morphological change before hatching). We considered 50% as the first stage in which it was possible to clearly distinguish the appearance of certain morphological features.

Animals at various stages of embryonic development (ED: 50%-60%-65.5%-70%-86%-94%-100%) were anesthetized by immersion in a 10% ethanol solution and then dissected to remove body wall tissues. All collected samples were processed for the experimental uses reported in the following paragraphs.

### Light microscopy and transmission (TEM) electron microscopy

Samples were collected and fixed with 4% glutaraldehyde in 0.1 M cacodylate buffer (pH 7.4) for 2–4 h at room temperature and then washed for 10 min, three times in the same buffer. Then, specimens were post-fixed for 1 h in 1% osmic acid in cacodylate buffer. After dehydration in an ethanol series, samples were embedded in an Epon–Araldite 812 mixture (Sigma-Aldrich) and sectioned with a Reichert Ultracut S ultratome (Leica, Nussloch, Germany). Semithin sections (0.70 µm) were stained by crystal violet and basic fuchsin and then observed with a light microscope (Eclipse Nikon, Amsterdam, Netherlands); images were acquired with a Nikon DS-SM camera. Thin sections (70 nm) were stained by uranyl acetate and lead citrate and observed with a Jeol 1010 electron microscope (Jeol, Tokyo, Japan).

### Immunogold staining at TEM

Samples were fixed for 2 h at 4 °C with 4% paraformaldehyde/0.5% glutaraldehyde in PBS, dehydrated in ethanol series, and embedded in an Epon-Araldite 812 mixture. Ultrathin sections were collected on gold grids (300 mesh, Sigma-Aldrich). After etching with 3% NaOH in absolute ethanol, they were incubated for 30 min in blocking solution containing PBS, 1% bovine serum albumin (BSA), and 0.1% Tween followed by treatment with a rabbit polyclonal anti-human RNASET2 primary antibody, diluted at 1:50 in blocking solution. After several washings with PBS, sections were incubated with the secondary goat anti-rabbit IgG (H+L) gold conjugate antibody (GE Healthcare, Amersham, UK; particle size, 10 nm) diluted at 1:40 in blocking solution for 1 h. In control experiments, the primary antibody was omitted. Sections were counterstained with uranyl acetate in water, observed with a Jeol electron microscope, and data were recorded with a digital camera system as previously described.

### Alcian blue staining

To highlight proteoglycans (PG), samples were fixed in 4% paraformaldehyde for 2 h and then washed three times in PBS buffer. Subsequently, tissues were dehydrated in an increasing scale of ethanol and paraffin embedded. Sections obtained with a paraffin microtome (7-µm thick) were stained with Alcian blue method. Briefly, sections were incubated for 30 min at room temperature in the staining solution (0.3% w/v Alcian Blue 8 GX in 3% acetic acid pH 2.5) and then differentiated with 3% acetic acid solution for 10 min. After washing, samples were treated with hematoxylin for 2 min, to counterstain nuclei. Images were recorded with an Eclipse Nikon microscope (Nikon, Tokyo, Japan).

### Immunofluorescence

All steps were performed at room temperature. Cryosections were rehydrated with PBS (pH 7.4) for 10 min and then pre-incubated for 30 min in blocking solution (2% bovine serum albumin and 0.1% Tween-20 in PBS, also used to dilute both the primary and the secondary antibodies). Samples were then incubated for 90 min with the primary antibodies (listed in Table 1), and after several washes in PBS buffer, they were incubated for 60 min with suitable secondary antibodies conjugated with cyanin 3 (Cy3, Abcam, dilution 1:400, Cambridge, UK). Nuclei were counterstained with 4′,6-diamidino-2-phenylindole (DAPI, 0.1 mg/mL in PBS) for 5 min and slides were mounted with Cityfluor (Cityfluor Ltd., London, UK). Negative control experiments were performed by omitting primary antibodies. Samples were finally observed under a fluorescence microscope (Eclipse Nikon) equipped with the emission filters 360/420 nm for DAPI nuclear staining and 550/580 nm for CY3 signals. Images were recorded with a Nikon digital sight DS-SM (Nikon, Tokyo, Japan) and mounted with Adobe Photoshop (Adobe Systems, San Jose, CA, USA).

**Table 1:** List of primary antibodies used for immunofluorescence (IF) and western blot (WB) studies

Antibody	Description	Company	Application	Dilution
Collagen Iα2	Rabbit polyclonal	Sigma-Aldrich	IF	1:200
			WB	1:500
Collagen IIIα1	Rabbit polyclonal	Proteintech	IF	1:100
			WB	1:500
Collagen IV	Rabbit polyclonal	Sigma-Aldrich	IF	1:100
			WB	1:500
RNASET2	Rabbit polyclonal	Kindly donated by Professor Acquati	IF	1:150
			WB	1:500
YAP1	Rabbit polyclonal	GeneTex	IF	1:150
GAPDH	Rabbit polyclonal	Proteintech	WB	1:7000

### HA (hyaluronic acid) staining

HA was detected by using a biotin-labeled HA-binding protein (HABP, Seikagaku Co, dilution 1:200), which recognizes HA saccharidic sequences. Sections were incubated with biotin-labeled HABP in blocking solution, overnight at 4 °C and, after washing, they were incubated for 1 h with streptavidin CY3-conjugated antibody (dilution 1:250) (Bertheim and Hellström, 1994).

Pairwise sequence alignments were performed to verify the homology between leech and human proteins (Table 2) and to validate the choice of the commercial antibodies used. In detail, both local and global alignments were conducted by means of EMBL-EBI software (https://www.ebi.ac.uk), in which the Needleman-Wunsch and the Smith-Waterman algorithms have been used, respectively.

**Table 2:** *H. verbana* and *H. sapiens* global sequence alignment. Both the identity and similarity values refer to the amino acid comparison and are reported as percentages

Protein	Lengths (aa)	Identity (%)	Similarities (%)
*H. verbana* Collagen Iα2	1388	45.6	54.7
*H. sapiens* Collagen Iα2	1366		
*H. verbana* Collagen IIIα1	1319	40.5	47.8
*H. sapiens* Collagen IIIα1	1163		
*H. verbana* Collagen IV	1745	45.9	54.0
*H. sapiens* Collagen IV	1669		
*H. verbana* YAP1	451	29.1	38.4
*H. sapiens* YAP1	504		

The local alignment between *H. verbana* and *Homo sapiens* Collagen Iα2, Collagen IIIα1, Collagen IV, and YAP1 protein sequences was also conducted to provide information about the conservation of the epitopes recognized by the antibodies used in the experiments (Supplementary Fig. 1).

### Western blot

Tissues obtained from embryos were immediately frozen in liquid nitrogen and then homogenized with a T10 basic ULTRA-TURRAX (IKA, Staufen, Germany) in 10 mL per mg of tissue of RIPA buffer (50 mM of NaCl, 1% NP-40, 0.5% sodium deoxycholate, 0.1% SDS, 50 mM of Tris–HCl, pH 7.5, protease/phosphatase inhibitor cocktail). The lysates were clarified by centrifugation (13,000 rpm at 4 °C for 20 min), and protein concentration was determined with the Bradford method (Serva, Heidelberg, Germany). Protein extracts were subjected to 8% SDS-PAGE (120-µg protein each lane); separated proteins were transferred onto 0.45-µm pore size nitrocellulose membranes (Amersham Protran Premium, GE Healthcare, Chicago, IL, USA). The filters were blocked for 2 h at room temperature with 5% (w/v) non-fat dried milk in TBS (Tris-buffered saline) and then incubated for 2 h at room temperature with the primary antibodies (Table 1) diluted in TBS/5% milk. After three washes of 10 min in TBST (Tris-buffered saline containing 0.1% Tween-20), the membranes were incubated for 1 h with horseradish peroxidase conjugated anti-rabbit secondary antibody (dilution 1:7500 in TBS/5% milk; Jackson ImmunoResearch Laboratories, West Grove, PA, USA). Finally, the membranes were exposed to the enhanced chemiluminescence substrate (LiteAblot PLUS, EuroClone), followed by autoradiography on X-ray film (KODAK Medical X-Ray film, Z&Z Medical, IA, USA). Densitometric analysis was assessed with the ImageJ software package 1. The values are reported as the relative optical density of the bands, normalized to glyceraldehyde 3-phosphate dehydrogenase (GAPDH), used as loading control.

About the detection of the housekeeping related to Collagen I, Collagen III, and Collagen IV the same membrane has been used, cutting it in two strips before the blocking; instead, about the detection of the housekeeping related to RNASET2, since they have a similar molecular weight, the expression of RNASET2 was firstly evaluated; then, labeled membrane was stripped in the stripping solution (62.5 mM Tris–HCl pH 6.8, 2% SDS, and 100 mM β-mercaptoethanol) at 50 °C for 30 min and, after washing with TBST, was reprobed with GAPDH antibody.

### RNA extraction and qPCR

Tissues extracted from adult or leech embryos at different development phases were instantly frozen in liquid nitrogen and homogenized with a mortar. The obtained homogenates were suspended in 1 mL of TRIzol reagent (Life Technologies) and incubated for 5 min at room temperature. Subsequently, 200 μL of chloroform was added and samples were centrifuged for 15 min at 13.000 rpm at 4 °C. Once the different phases separated, 500 μL of the supernatant was recovered and gradually mixed with 500 μL of isopropanol and centrifuged for 10 min at 13.400 rpm. Then, 1 mL of EtOH 75% diluted in DEPC water was added to the precipitated RNA pellet that, after a subsequent centrifugation, was finally resuspended in 30 μL of DEPC water and incubated for 10 min at 55 °C. Putative contaminating DNA was removed by a TURBO DNA-free kit (Thermo Fisher Scientific) and samples were quantified, with purity being evaluated on 1% agarose gel. In total, 2 μg of RNA were retro-transcribed into cDNA using M-MLV reverse transcriptase (Life Technologies) and qPCR was conducted in triplicate with the iTaq Universal SYBR Green Supermix (Bio-Rad, Hercules, CA) using a 96-well CFX Connect Real-Time PCR Detection System (Bio-Rad). The primers used for qPCR amplifications are listed in Table 3.

**Table 3:** List of primers used for quantitative PCR analysis

Target gene	Primers	Product size (bp)
Collagen Iα2	Fw: 5′-AAGGGAGAGCAAGGAAGACA-3′	158
	Rv: 5′-CCTG GTAAGCCATCAACACC-3′	
Collagen IIIα1	Fw: 5′-GGGACCTTCAGGCGATAGAG-3′	71
	Rev: 5′-TCTCTCCTGACTTGCCCTGA-3′	
Collagen IV	Fw: 5′-CCTCCTAACACTACAGCCCT-3′	85
	Rev: 5′-TGCCTAAATCTTGCGTTGCT-3′	
RNASET2	Fw: 5′-GGTCCCAA CTTCTGCACAAAGGAT-3′	136
	Rev: 5′-GTTTGTCCCATTCATGCTTCCAGAA-3′	
YAP1	Fw: 5′-ACCAGTCATCAGCACTACCA-3′	119
	Rev: 5′-TGAACAGCAAGTCCAACTCG-3′	
GAPDH	Fw: 5′-GAAGACTGTGGATGGACCCT-3′	121
	Rev: 5′-GTTGAGGACTGGGATGACCT-3′	

To calculate the relative gene expression, the 2–ΔΔCt method was used with GAPDH considered as housekeeping gene. After an initial denaturation phase, qPCR was performed at 95 °C (10 s), 60 °C (5 s), and 72 °C (10 s) for 39 cycles. Graphs show the COL1α1, COL3, COL4, HvRNASET2, and YAP1 quantification (fold change) relative to the GAPDH gene expression.

### Statistical analyses

Western blot and qPCR experiments were conducted in triplicate and data represent the mean values ± SEM. Statistical analyses were performed using GraphPad Prism 7 (GraphPad Software, La Jolla, CA, USA). Statistical differences were calculated by one-way ANOVA followed by Tukey’s post hoc test, and p<0.05 was considered statistically significant. In the assays, means with different letters correspond to the significant differences among different developmental stages.

## Results

### Animal model description

The body wall of an adult *Hirudo verbana* is described in Supplementary Fig. 2. The final body organization of adult leeches is reached 3–4 months following hatching. During this period, the most significant differences between newborns and adults are related to changes in body size, due to a massive numerical and dimensional increases of muscle fibers, coupled to the reorganization of the ECM (Pulze et al. 2022).

Here, we report a detailed description of leech’s embryogenesis in order to complete the complex picture of the events that occur in the formation of the muscle cutaneous sac that underlie the typical motility of these animals.

### Morphological analyses at optical and electron microscope (TEM) of leech embryos

Compared to newly hatched leeches, the organization of the embryos’ bodies developing inside the cocoon was deeply different, despite a gross phenotypical similarity.

**Fig. 1:**
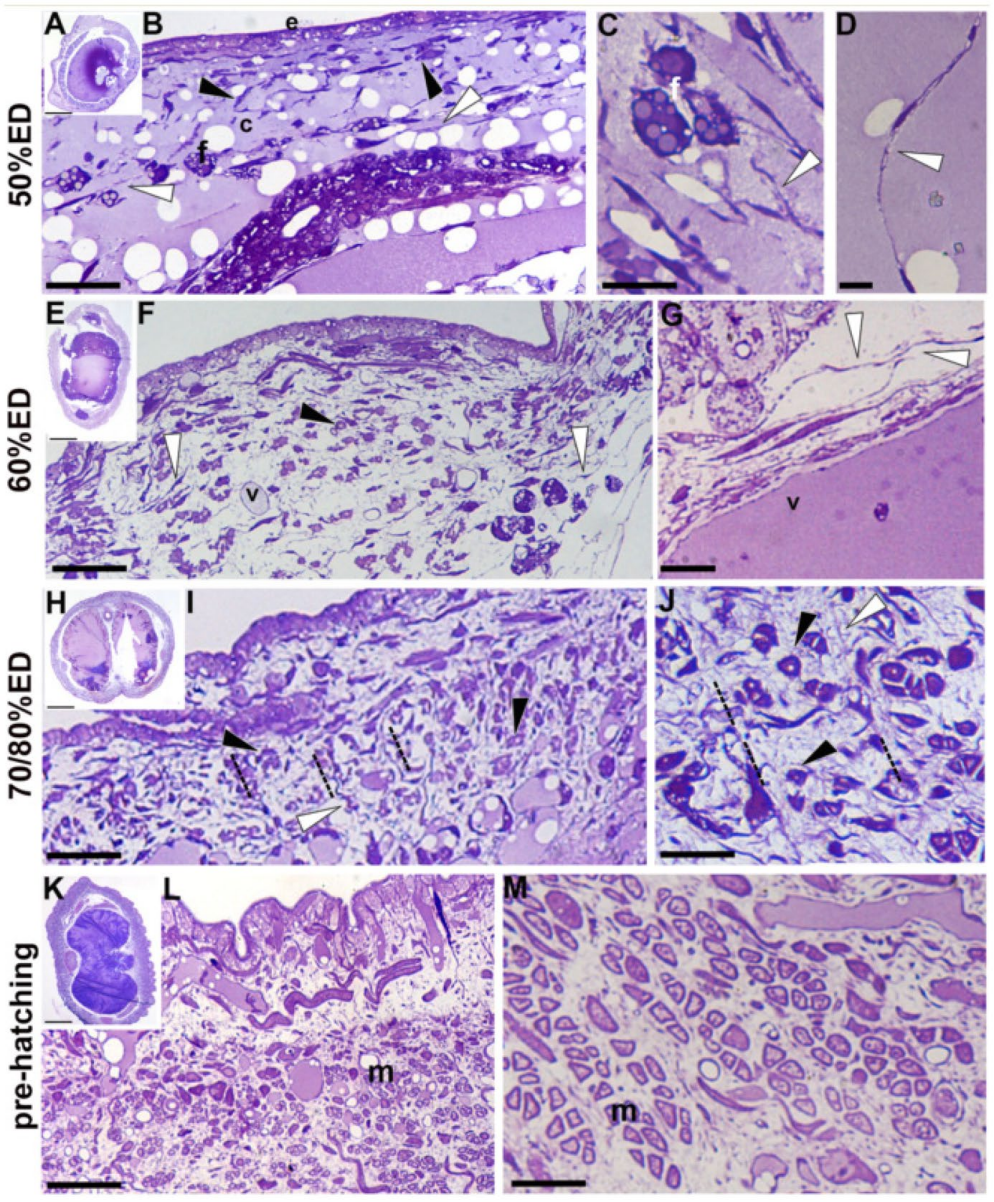
Semithin sections stained with crystal violet and basic fuchsin. **A**–**M** General view of cross-sectioned body of 50% ED, 60% ED, 70/80% ED, and pre-hatching of *H. verbana* extracted from the cocoon. **A**, **B** In 50% ED stage, the thin body wall is mainly composed of loose connective tissue. A monolayered epithelium (e) surrounds few cells (black arrowheads), fibroblasts (f), and TCs (white arrowheads) that are embedded in abundant ECM (c). **C** Fibroblasts (f), showing the cytoplasm filled with lipid droplets, are in close contact with the long cytoplasmic expansion of TCs (arrowheads). **D** TCs (white arrowhead) with their long telopodes. **E**, **F** In 60% ED stage, the thickness of the body wall increases due to numerous cells, sometimes grouped (black arrowheads) and embedded in the ECM. TCs (white arrowheads), forming a dense web-like, are visible. **G** Detail of TCs, showing their long telopodes (white arrowheads), close to a blood vessel (v). **H**, **I** The body wall of 70/80% ED embryos is thicker and small cluster of cell (encircled) spatial organized can be noted. **I**, **J** Corridors (dotted lines), bordered by TCs (white arrowheads), occupied by small muscle fibers (black arrowheads) are visible. **K**, **L** Pre-hatching leeches are more similar to the newborns. The muscle fibers, very few and small in size (m), are embedded in a loose ECM. **M** Detail of muscle fibers (m) with the contractile material surrounding a cytoplasmic core. Scale bars: **A**, **E**, **H**, **K** 400 μm; **B**,** F**,** I**,** L** 30 μm; **C**,** D**,** G**,** J**,** M** 10 μm

Starting from 50% ED (the first embryonic stage with a consistency allowing experimental manipulation) up to pre-hatching stage, we observed a progressive increase in the thickness of the body wall, due to the visible increase in cell number that appeared to be inversely proportional to the synthesis of connective tissue, as already reported in post embryonic development (Pulze et al. 2022), (Fig. 1). Of note, the key differences among distinct embryos’ developmental stages were mostly related to the cell types forming the body wall, their differentiation pattern, spatial distribution, and ECM organization.

In 50% ED embryo samples, the thin cuticle and the monolayered epithelium underneath enveloped a loose connective tissue containing few cells, isolated or in contact with each other (Fig. 1A, B). Among them, fibroblasts with lipid droplets in the cytoplasm (Tettamanti et al. 2004) and TCs, characterized by long telopodes, were well recognizable (Fig. 1C, D).

In embryos at around 60% ED, characterized by reduced and uncoordinated body movements, the body wall appeared thickened, due to an increased connective tissue deposition, and filled with different type of scattered distributed cells (Fig. 1E–G), among which TCs were highly represented (Fig. 1G).

From 70/80% ED, the embryos started to showed active movements and their body wall structure appeared radically changed (Fig. 1H, I), due to the massive increase in the number of cells spatially organized in small clusters. At higher magnification, these cells appeared to be tiny cyrcomiarian helical muscle fibers, with the contractile material encircling a central cytoplasmic core. These muscle fibers were mainly localized in corridors delimited by TCs (Fig. 1J). In pre-hatched embryos (90–100%), groups of well-differentiated muscular fibers, still delimited by TCs, were clearly distinguishable (Fig. 1K–M). In this analysis, we thus reported the occurrence of a tight proximity between muscle fibers and TCs throughout the different developmental stages investigated.

**Fig. 2:**
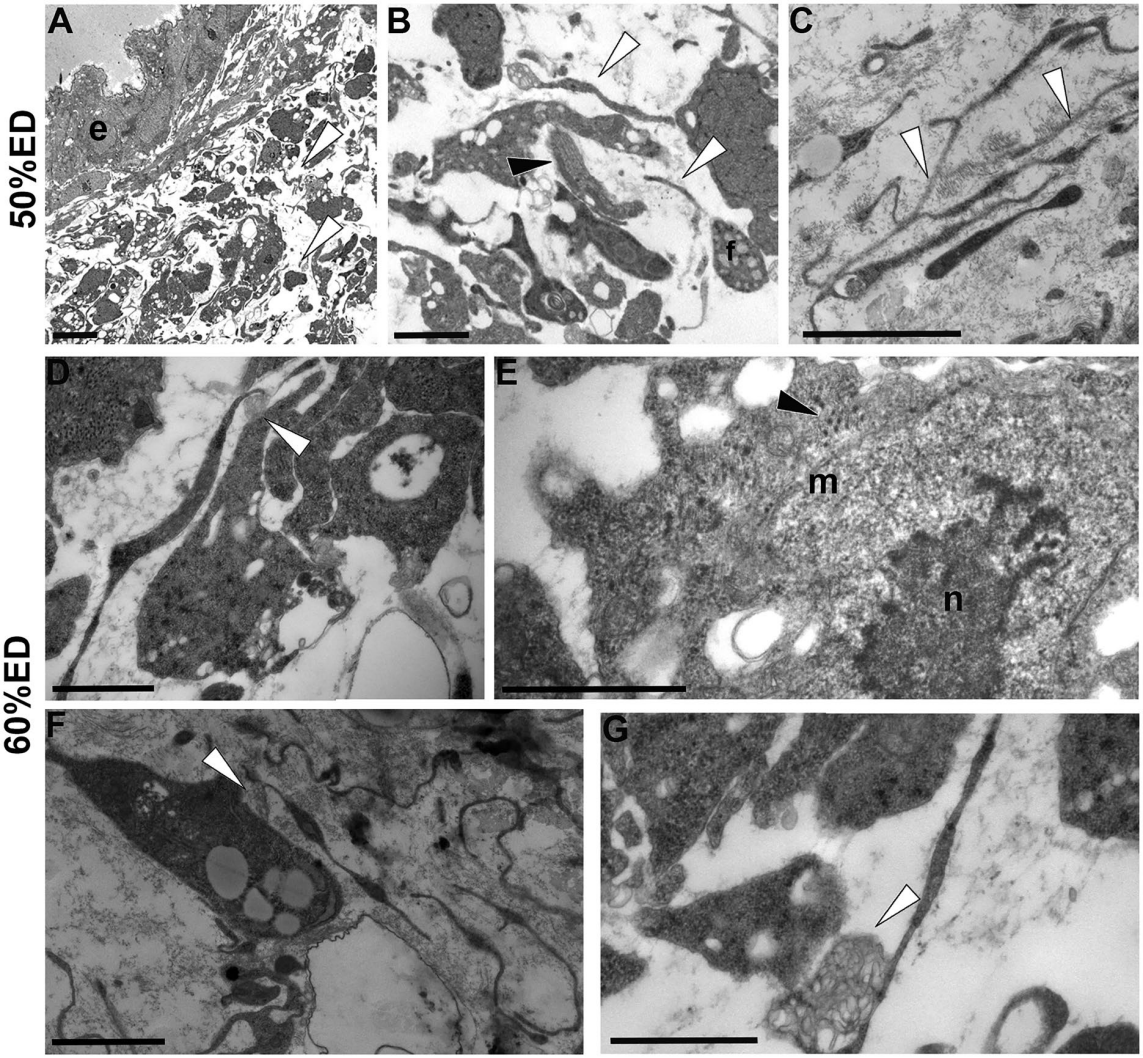
Transmission electron microscopy (TEM). **A**–**G** General view and details of cross-sectioned body of 50% ED and 60% ED of *H. verbana* extracted from the cocoon. **A** In the 50% ED stage, under the thin epithelium (e) few cells, scattered in the loose ECM, form the body wall. **B** Detail of blast-like cells (black arrowhead) and, among them, TCs (white arrowheads) are shown. **C** Detail of TCs showing long interconnected telopodes (white arrowheads). **D**–**G** In 60% ED embryos, among cells embedded in ECM, poorly differentiated myocytes show few contractile material (black arrowhead) (**D**, **E**). Numerous multivesicular bodies (white arrowheads) are close to target cells, both myocytes (**D**, **G**) and fibroblasts (**F**). Scale bars: **A** 5 μm; **B**, **C**, **F** 2 μm; **D**, **E**, **G** 1 μm

Ultrastructural analysis of 50% ED embryos confirmed that, beneath the epithelium, the ECM was mainly populated by scattered, undifferentiated blast-like cells (de Eguileor et al. 1993, 1998), characterized by a high nucleus-to-cytoplasm ratio and interconnected by long TCs’ telopodes, forming a 3D network within an abundant connective tissue (Fig. 2A–C).

In 60% ED embryos, for the first time, few contractile elements were evident in the cytoplasm of blast cells differentiating in primary myocytes (Fig. 2D, E). Of note, multivesicular bodies produced by TCs’ telopodes were found in close association with both fibroblasts (Fig. 2F) and differentiating myocytes (Fig. 2G).

**Fig. 3:**
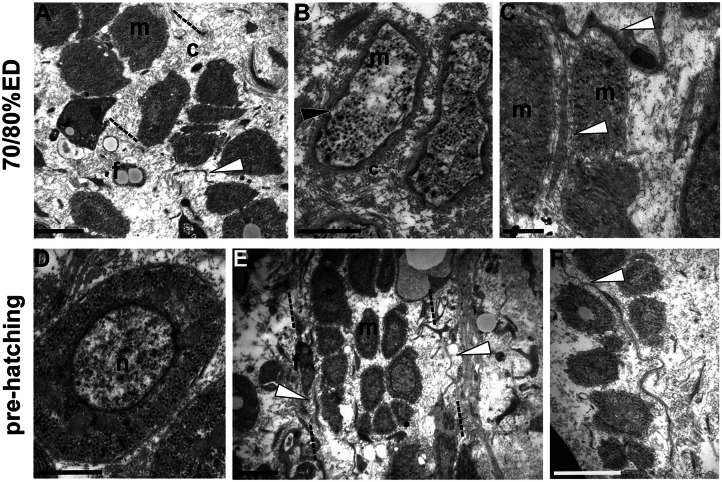
Transmission electron microscopy (TEM). **A***–***F** General view and details of cross-sectioned body of 70/80% ED and pre-hatching of *H. verbana* extracted from the cocoon. At this developmental phase, grouped myocytes (m) and fibroblast (f) migrate, from the center towards the surface of the body, in corridors (identified by dotted lines) delimitated by TCs (white arrowhead), and characterized by loose ECM (c) (**A**). Myocytes (m), with disorganized contractile material (black arrowhead), are small in size and display a migrating phenotype (**B**). **C** Detail of a TC interposed between two myocytes. In pre-hatching stage, muscle fibers show a well-organized contractile material that surrounds a cytoplasmic core occupied by large nucleus (n). In cross-sectioned fibers, 4 to 5 series of myosin and actin filaments, organized in “sarcomeres,” already defined by short Z elements, are evident (**D**). Besides muscles, connective tissue is populated also by fibroblasts and other cellular types, and by resident cells, the TCs (white arrowheads). TCs delimit corridors (**E**) and communicate with other cells (**F**). Scale bars: **A**, **E**, **F** 2 μm; **B**, **D** 1 μm; **C** 500 nm

Starting from 70/80% ED up to pre-hatching stage (90–100%ED), cyrcomiarian helical muscle fibers, with contractile material arranged in a thin peripheral ring, were clustered in well-defined groups, still delimited by TCs (Fig. 3A–F).

### Characterization of ECM components

**Fig. 4:**
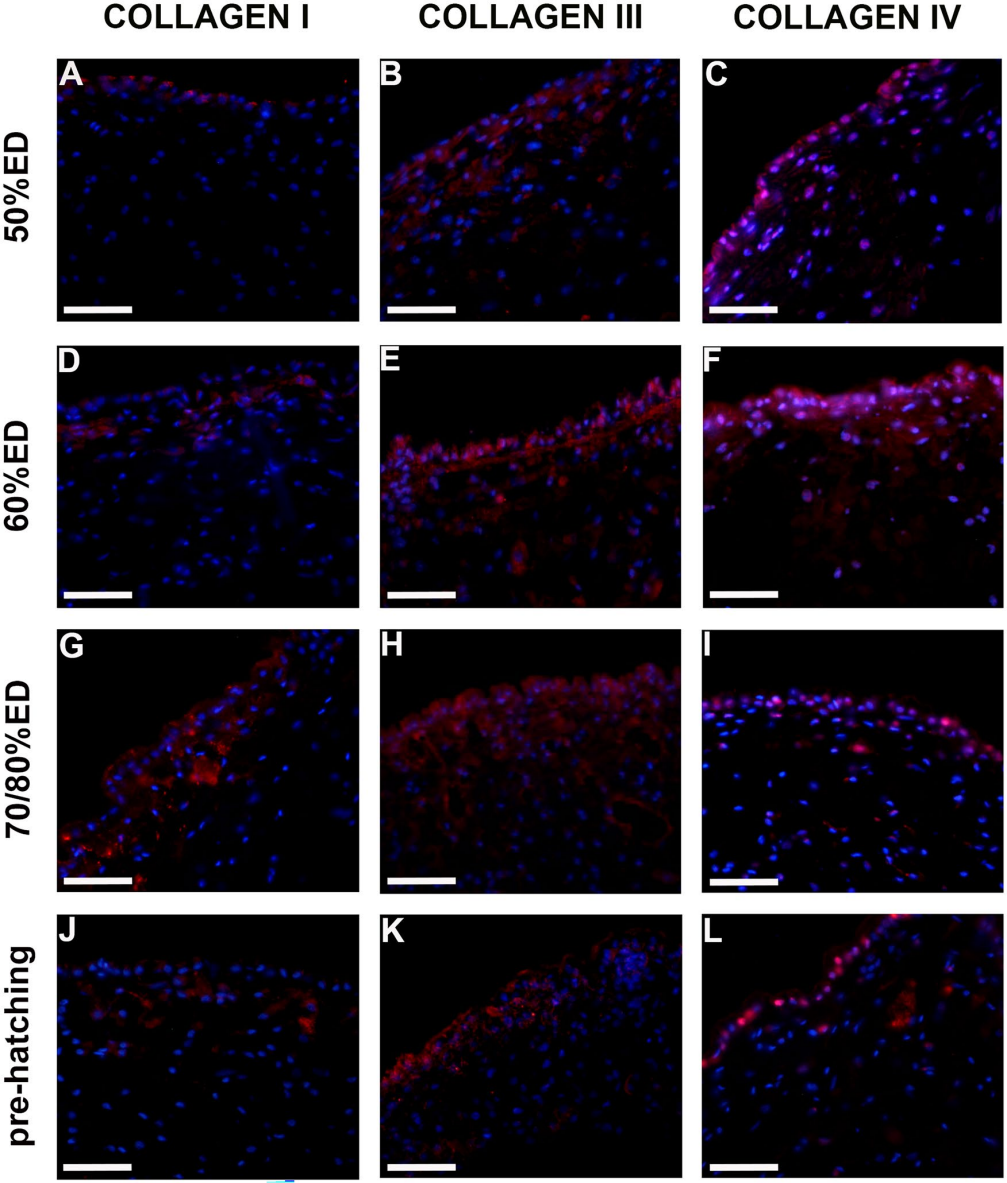
Immunocharacterization of collagens in cross-sectioned body of 50% ED, 60% ED, 70/80% ED, and pre-hatching of *H. verbana* extracted from the cocoon (**A**–**L**). Immunofluorescent analyses show many cells positively reacting with Collagen I (**A**, **D**, **G**, **J**), III (**B**, **E**, **H**, **K**), and IV (**C**, **F**, **I**, **L**) antibodies (in red). Collagens I and III are present in larger quantity in 70/80% ED in respect to other developmental phases, while the signal for the non-fibrillar Collagen IV is higher during the 60% ED stage. Nuclei are counterstained with DAPI (in blue). Scale bars: **A**–**L** 40 μm

**Fig. 5:**
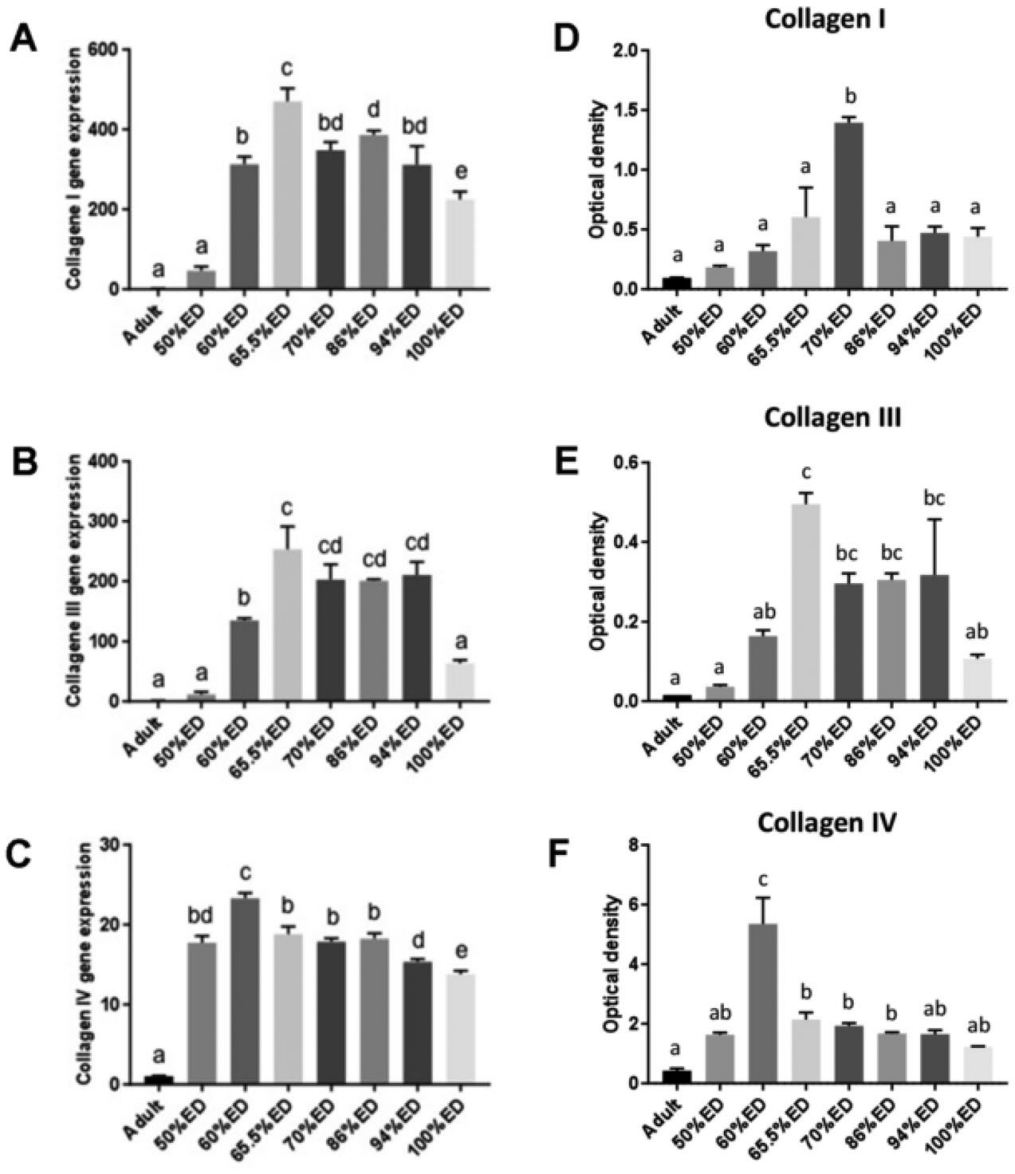
Collagen I, III, and IV qRT-PCR and western blot analyses of adult, 50% ED, 60% ED, 70/80% ED, and pre-hatching of *H. verbana* extracted from the cocoon. The graphs in the left column show Collagen I (**A**), III (**B**), and IV (**C**) mRNA expression in different embryonic developmental stages and also in adult leech (where expression levels are already known). Collagen values were normalized with the expression of the endogenous gene glyceraldehyde 3-phosphate dehydrogenase (GAPDH). The graphs in the right column represent protein expression levels of Collagen I (**D**), III (**E**), and IV (**F**) in the same stages. The data result from a densitometric analysis of the western blots. The values are reported as relative optical density of the bands normalized to GAPDH. Statistical differences were calculated by one-way ANOVA followed by Tukey’s post hoc test; error bars represent SEM and different letters denote statistically significant differences (*p* < 0.01)

During leech’s embryogenesis, three types of collagens (I, III, and IV) were detected by immunofluorescence (Fig. 4A–L), qPCR, and immunoblotting assays (Fig. 5).

Even though the fibrillar Collagens I and III and the non-fibrillar Collagen IV (the latter constituting the backbone of basement membranes) were highly expressed during all embryonic developing stages, Collagens I and III reached a peak of expression at 70% ED, while Collagen IV peaked at 60% ED, as also confirmed by molecular analyses (Figs. 4 and 5).

**Fig. 6:**
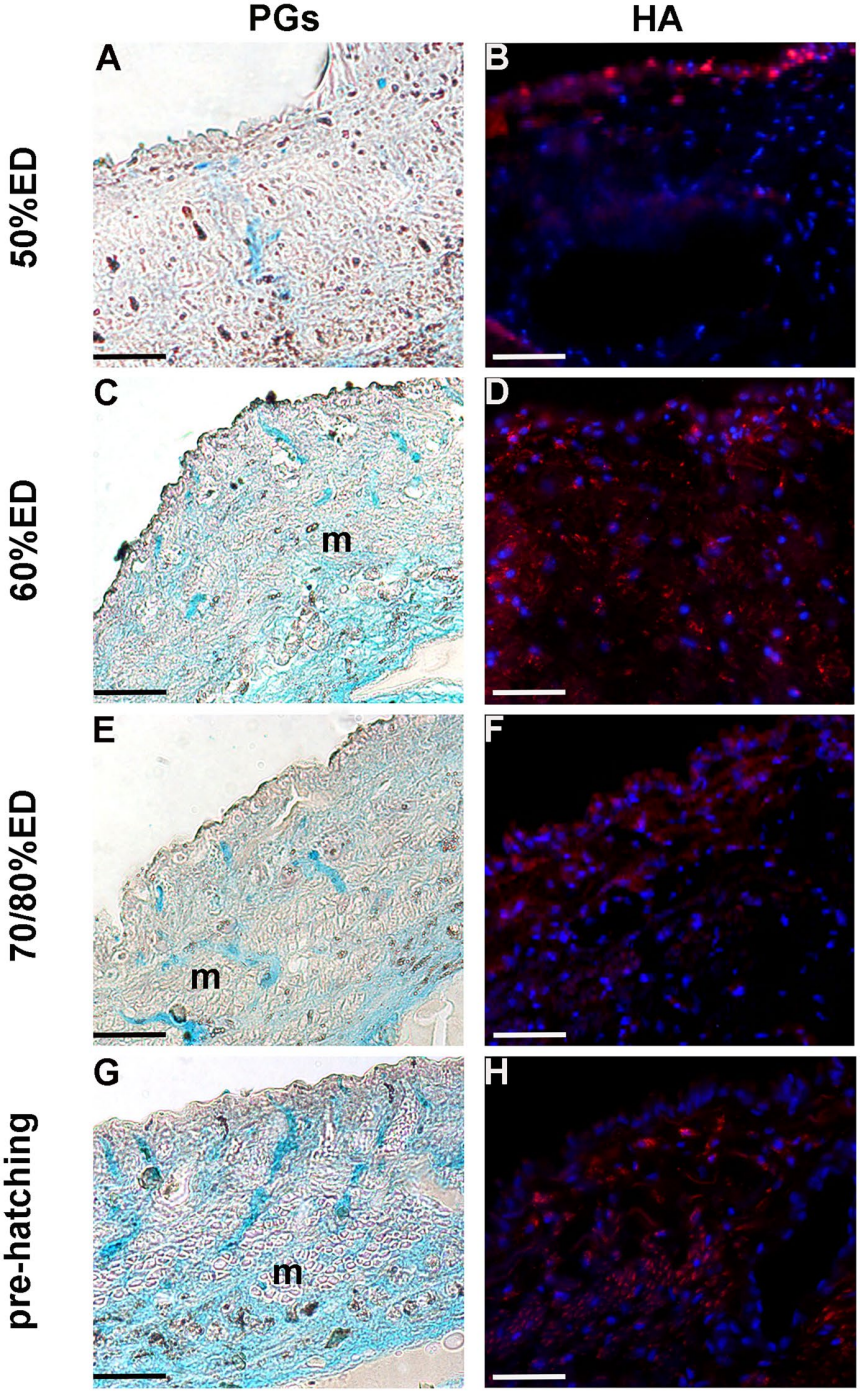
Analysis of PGs in 50% ED, 60% ED, 70/80% ED, and pre-hatching of *H. verbana* extracted from the cocoon. Proteoglycans are highlighted (green–blue staining) with Alcian blue method (**A**, **C**, **E**, **G**), while the staining of HA (red) is performed using an HA binding protein (**B**, **D**, **F**, **H**). Nuclei are counterstained respectively with hematoxylin and DAPI. Scale bars: **A**–**H** 40 μm

As in vertebrates, proteoglycans (PGs) are composed of a core protein and GAG chains [particularly hyaluronan (HA)] in leeches as well and represent important components of the ECM (Tettamanti et al. 2005; Pulze et al. 2022). Our data showed that both PGs and HA were present in the ECM during all phases of embryonic development and their amount was particularly abundant at 60% ED stage, as assessed by conventional procedures (Fig. 6A–H).

### YAP1 detection

**Fig. 7:**
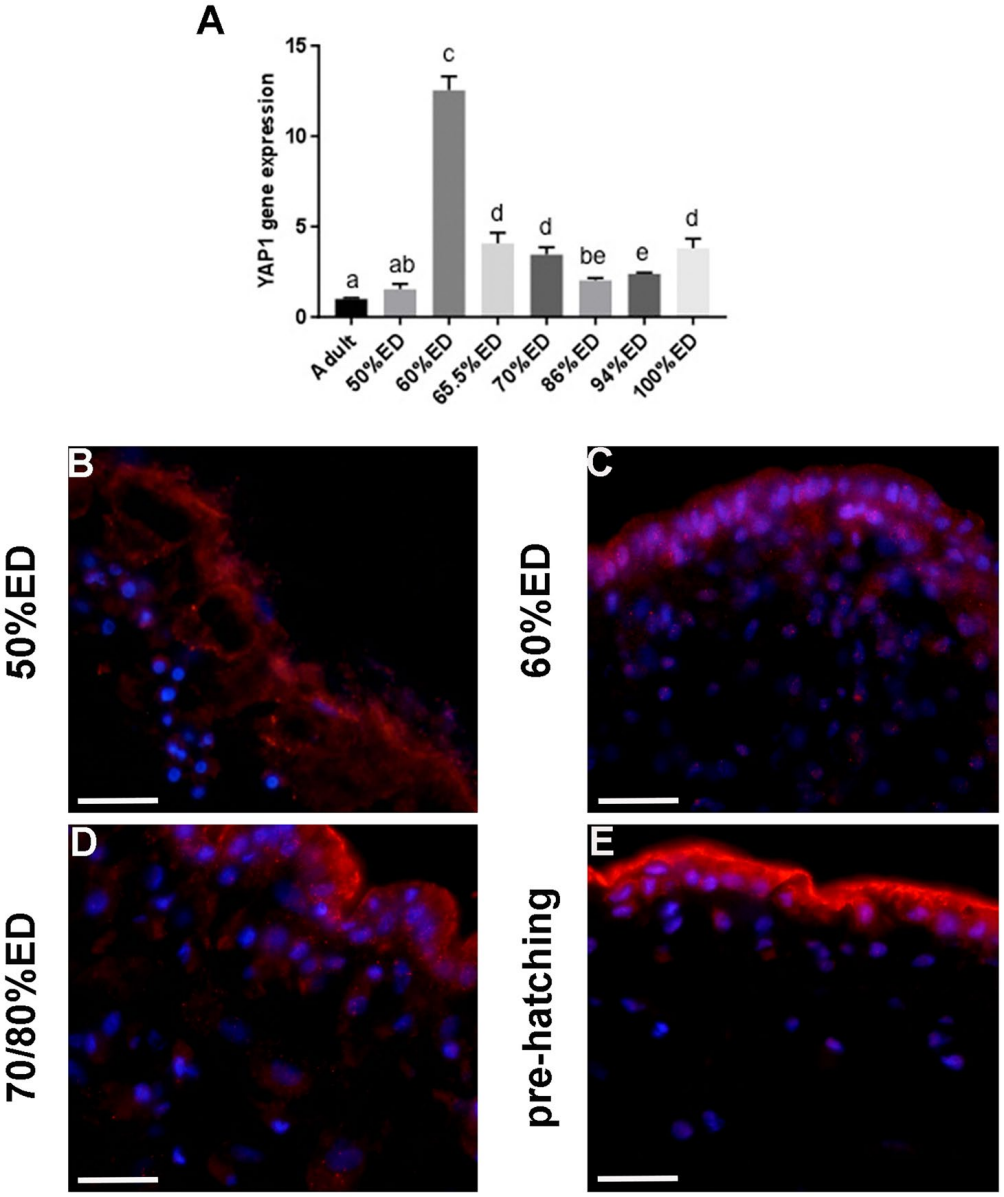
qRT-PCR analyses and immune-characterization of YAP1. Graph **A** shows YAP1 mRNA expression in different embryonic developmental stages and also in adult leech (where expression levels are already known). The values are normalized with the expression of the endogenous glyceraldehyde 3-phosphate dehydrogenase gene (GAPDH). Statistical differences were calculated by one-way ANOVA followed by Tukey’s post hoc test; error bars represent SEM and different letters denote statistically significant differences (*p* < 0.01). Immunolocalization of YAP1 (red signal) reveals the subcellular localization of the protein, which is preferentially expressed in the nuclei of the 50% ED, 60% ED, and pre-hatching stages (**B**, **C**, **E**) and also in the cytoplasm of the 70/80% ED stage (**D**). Nuclei are counterstained with DAPI. Scale bars: **B**–**E** 20 μm

Our previous studies pointed at YAP1 as a transcriptional regulator with pivotal roles on cell differentiation during leech post-embryonic development through a mechanosensing process (Pulze et al. 2022). Here, we speculate that YAP1 might be required in the modulation of cell differentiation during leech embryonic development. Indeed, qRT-PCR analysis showed that YAP1 transcript expression increased progressively starting from 50% ED, reaching a peak at 60% ED (Fig. 7A). Interestingly, the highest level of YAP transcript occurred just before the peak of expression of fibrillar Collagens I and III and PGs, both responsible for the changes in ECM remodeling.

Immunofluorescence staining showed that in all developmental stages analyzed, YAP1 was preferentially localized in cell nuclei (Fig. 7B, C, E). However, at 70/80% ED, it was also detected in the cytoplasm of some cells throughout the developing body wall (Fig. 7D), confirming its involvement in myocyte differentiation, as morphologically described in the same stage.

### HvRNASET2 detection

**Fig. 8:**
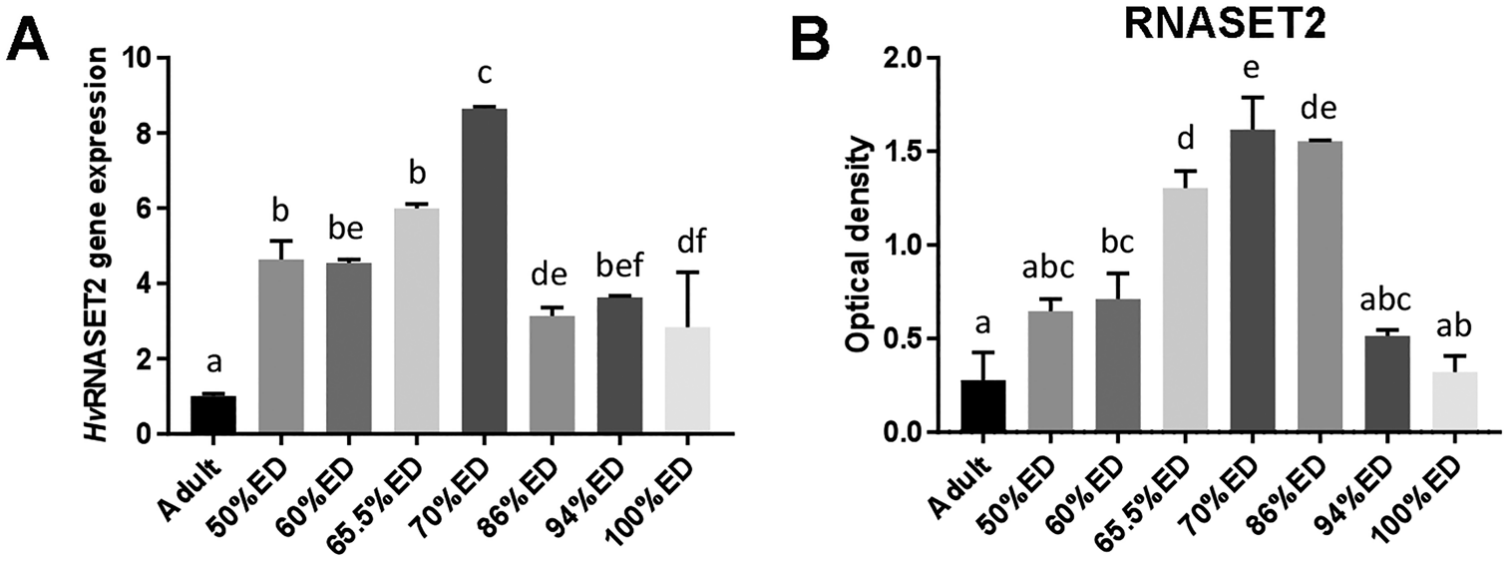
*Hv*RNASET2 qRT-PCR and western blot analyses of adult, 50% ED, 60% ED, 70/80% ED, and pre-hatching of *H. verbana* extracted from the cocoon. Graph **A** illustrates *Hv*RNASET2 mRNA expression in different embryonic developmental stages and also in adult leech (where expression levels are already known). The values are normalized with the expression of the endogenous glyceraldehyde 3-phosphate dehydrogenase (GAPDH) gene. Graph **B** represents *Hv*RNASET2 protein expression in the same stages. The data result from a densitometric analysis of the western blots. The values are reported as relative optical density of the bands normalized to GAPDH. Statistical differences were calculated by one-way ANOVA followed by Tukey’s post hoc test; error bars represent SEM and different letters denote statistically significant differences (*p* < 0.01)

**Fig. 9:**
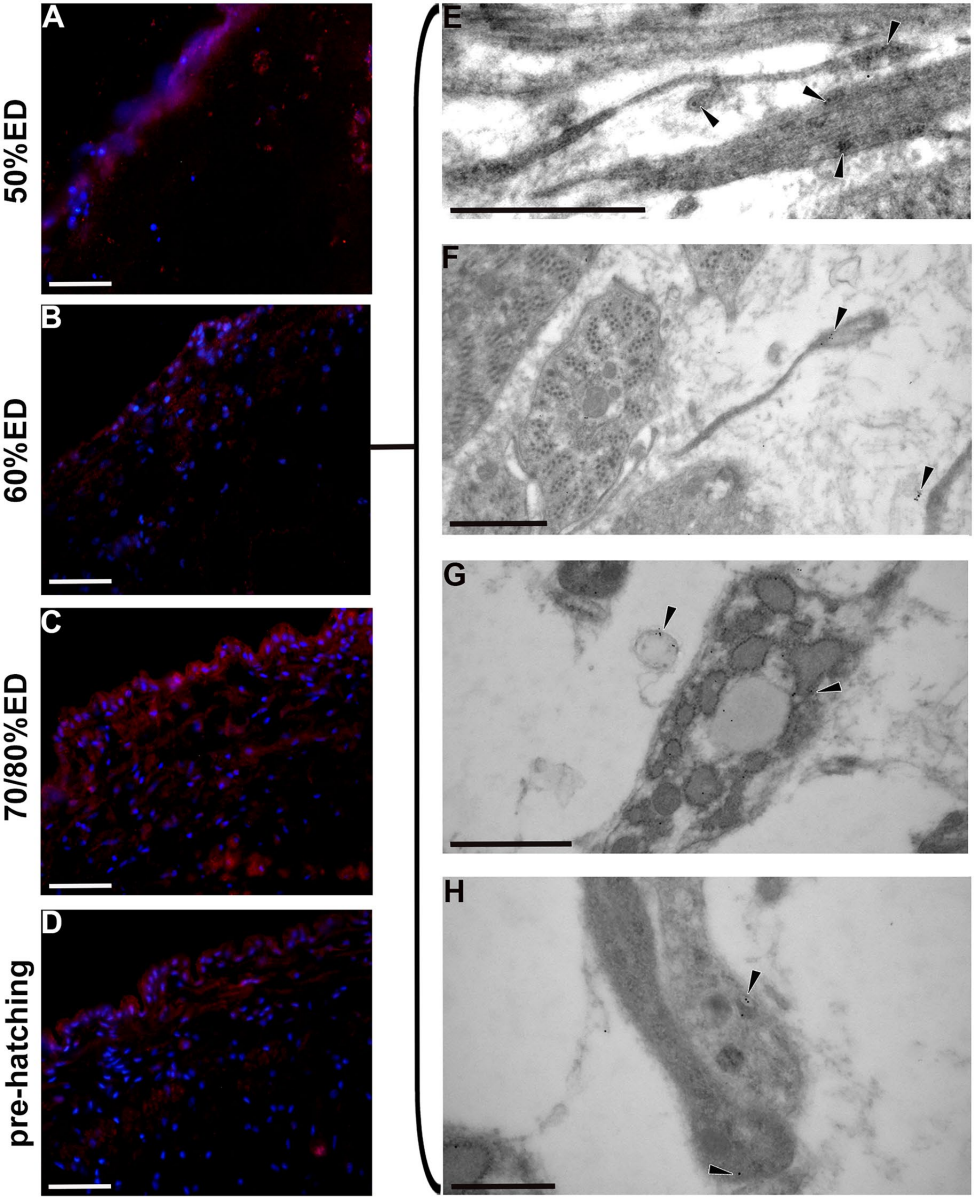
Immunocytochemical and ultrastructural analysis of *Hv*RNASET2 in cross-sectioned body of 50% ED, 60% ED, 70/80% ED, and pre-hatching of *H. verbana* extracted from the cocoon.** A**–**D** Immunolocalization of *Hv*RNASET2 (red) confirms the expression in all the stages analyzed, above all in 70/80% ED stage (**C**). Nuclei are counterstained with DAPI. **E**–**H** Immunogold analyses. HvRNASET2 (black arrowheads) is visualized closely associated with shedding (**E**), synthetizing TCs (**F**–**H**), and in multivesicular bodies (**G**). Scale bars: **A**–**D** 40 μm; **E**–**G** 1 μm; **H** 500 nm

As already reported, *Hv*RNASET2 is able to induce the recruitment and proliferation of fibroblasts and triggers a massive remodeling of connective tissue during leech wound healing and tissue regeneration (Baranzini et al. 2021). Based on the hypothesis that tissue regeneration and embryonic development both rely on similar processes (Karalaki et al. 2009), we evaluated the correlation between *Hv*RNASET2 expression and ECM remodeling during leech embryonic development. To this aim, we performed both molecular and immunohistochemical assays. qRT-PCR and western blot analyses showed that *Hv*RNASET2 was expressed at all developmental stages, with an expression peak observed at 70% ED (Fig. 8A, B). Immunofluorescence assay confirmed that *Hv*RNASET2 was detectable in all steps of embryonic development, showing the same expression trend previously observed in molecular analyses (Fig. 9A–D). Of note, immunogold analysis highlighted the presence of *Hv*RNASET2 in correspondence of TCs and exocyted vesicles (Fig. 9E–H).

## Discussion

By thoroughly monitoring the development of *H. verbana* from hatching to the adult stage, we have shown that the leech body wall organization deeply changes in terms of thickness, number of muscle cells, degree of differentiation, and ECM organization. However, in spite of these differences, differently staged leeches showed phenotypical and morphological similarities. As expected, changes in the stiffness of the ECM were largely attributable to the massive production of collagens.

A key question addressed in this study is how a complex muscle mass comes to be organized in a particular, highly stereotyped fashion during *H. verbana* development. The proper organization of the structural plan (in particular the muscle’s identity) is crucial for the expression of behavioral roles in newborn leeches, where positioning of muscles appears to be governed by precise mechanisms leading to highly ordered arrangements.

The body wall organization of *H. verbana* embryos during development within the cocoon was shown to undergo abrupt changes from the first stage that can be examined (about 50% ED) up to the pre-hatching (90–100%), when embryos actively move and push against the interior wall of the cocoon.

At first, the leech body is made of a thin epithelial sac wrapping few cells and small organs embedded in loose connective tissue. At the time of hatching, a thicker muscular cutaneous sac and a concomitant change in the level of inner organization are observed, due to the development of different complex organs.

The intra-cocoon architecture of embryos evolves through several steps. In early embryos (50–60% ED) the leech body is frail, made of a thin epithelium and cuticle enveloping abundant ECM, few cells with blast-like phenotype, fibroblasts, few isolated primary myocytes characterized by disorganized contractile material, and TCs that are the most represented cell type.

TCs, already described in adult leeches (Pulze et al. 2017), are interstitial cells characterized by thin and long cytoplasmic processes, called telopodes, which exhibit a distinctive moniliform shape and often follow a sinuous trajectory. Telopodes, within the stromal space, typically organize intricate networks that provide a structural support and a guide for tissue organization, as previously demonstrated in vertebrate morphogenesis (Bani et al. 2010; Díaz-Flores et al. 2013; Kondo and Kaestner 2019). Thus, embryonic TCs, acting as a 3D mechanical support, provide a stiff structure that is resistant to deformation and thus supportive for cell migration. Furthermore, the long cytoplasmic processes of TCs are critically involved in communication with neighboring cells, by means of specialized cell-to-cell junctions (gap junctions) (Gherghiceanu and Popescu 2012; Cretoiu et al. 2013; Pulze et al. 2017) and by the release of regulatory paracrine signaling molecules (Cretoiu et al. 2015).

Starting from the earliest stages and during the entire embryonic life of leech development, TCs are thought to represent a source of regulatory paracrine signals (among which *Hv*RNASET2 might play a key role) that are delivered to neighboring cells through exosomes. We hypothesize that *Hv*RNASET2, working in short range communications to regulate fibroblast activation and collagen production (Baranzini et al. 2017, 2019, 2020a, b), might also be involved in the massive connective tissue remodeling during leech embryonic development. Indeed, once activated by *Hv*RNASET2, fibroblasts are engaged in the massive production of fibrillar and non-fibrillar collagens [46]. In this context, types I and III collagen fibers, typically anisotropic and curly, work as “filler material” supporting the primitive scaffold of TCs. Collagens I and III are co-expressed and represent the main ECM components during the entire intra-cocoon life, with an expression peak around 70% ED. Collagen type IV, as a backbone of basement membrane, is also largely expressed during leech development (as reported in vertebrate morphogenesis) with an expression peak at 60% ED (Rozario and DeSimone 2010) and might provide a structural support involved in the ensuing separation of embryonic tissues into compartments and in the modulation of cell behavior. Furthermore, PGs and GAGs, such as HA, being highly expressed in interstitial matrix (as shown by histo- and immunocytochemical techniques) and aggregating with other molecules, might be involved in providing tissue resilience. The abundance of these molecules that, as in vertebrates, are critical in the regulation of cell proliferation and migration could also be involved in water retention to create a kind of fluid skeleton that might serve as a support for both cell locomotion and tissue organization, so important in soft-body animals without true cavities.

During the intra-cocoon life, the arrangement of leech muscle cells that will later become the predominant type of cells in the body wall does not proceed randomly but rather shows a precise pattern which change from the earliest phases, as observed for vertebrate skeletal muscle. Such organization is important to ensure the efficient morphogenetic events leading to the increased organization level within the leech body.

In this scenario, we suggest that the formation of a 3D scaffold of cells and ECM triggers the spatiotemporal events modulating the embryo’s architecture and patterning. Indeed, at about 70% ED, TCs’ and ECM networks template the body wall muscle organization and very small muscle fibers start to display a spatially defined pattern organized in small groups. Meanwhile, the ECM remodeling is correlated to collagen neo-synthesis and fibroplasia events, as demonstrated by immunocytochemical data. TCs, as resident cells and concealed in the ECM, might actively participate to this process by guiding myogenic progenitor cells and myocytes during their differentiation and spatial organization in leech body wall.

As long as the ECM is concerned, it is important to underline that, besides providing structural support and sequestering or storing signaling molecules, it also senses and transduces mechanical signals, as already demonstrated in post-hatching life (Pulze et al. 2022). One of the most interesting findings emerged from our studies on *H. verbana* development from hatching to adult pointed at YAP1, a transcriptional regulator, as a key molecular effector playing critical roles in mechanotransduction, organ development, and regeneration. According to the level of ECM stiffness, YAP/TAZ translocate in the nucleus to maintain the stemness state or remain in the cytoplasm to promote cell differentiation (Totaro et al. 2018; Cai et al. 2021). Besides these functions, YAP/TAZ, as in vertebrates, could modulate fibroblast proliferation (Mia et al. 2022) and in turn ECM remodeling by cooperating with the recently discovered leech molecule *Hv*RNASET2 (Baranzini et al. 2017, 2019, 2020a, b), a regulator of ECM remodeling during wound healing that acts by inducing the activation of fibroblasts and promotes the synthesis of new Collagen I. Considering that regeneration recapitulates some of the processes underlying embryonic development, we speculate that *Hv*RNASET2 might represent a key signaling molecule involved not only in the restoration and maintenance of tissue homeostasis during muscle regeneration but also in the remodeling of the extracellular matrix during muscle development.

Indeed, during early leech embryogenesis, starting from 50 up to 70%, *Hv*RNASET2 appears to be released from TCs via exosomes and might be involved in the modulation of proliferation and activation of collagen-producing fibroblasts. Indeed, as demonstrated by molecular analyses, *Hv*RNASET2 and fibrillar collagens show overlapping dynamics in their expression pattern. Interestingly, YAP1 expression reaches a peak during earlier developmental stages. These interconnections suggest not only the highly dynamic role of the ECM in migration, differentiation, and spatial organization of cells but also point at the interplay between ECM and those cells that can in turn affect gene expression responding to mechanical signals. Indeed, immunofluorescence assays clearly showed that the specific subcellular localization of YAP1 changes was correlated with ECM remodeling occurring during embryonic leech development.

Taken together, this work provides a preliminary picture of the cellular and extracellular effectors that might play a key role in the regulation of leech development, with particular attention paid to the muscle tissue.

Although further functional studies are no doubt needed, we reckon that our data lay the foundations to better investigate the molecular and cellular effectors involved in key stages of *H. verbana* development.

### Supplementary Information

Below is the link to the electronic supplementary material.

Supplementary file1 (PPTX 62 KB)

Supplementary file2 (DOCX 406 KB)
