# Supplementary figures and images for: Dynamic relationship among extracellular matrix and body wall cells in Hirudo verbana morphogenesis
Source: Cell Tissue Res. 2024 Mar 1;396(2):213–29. doi: 10.1007/s00441-024-03874-x (PMC11055932; doi:10.1007/s00441-024-03874-x)

## Slide 1
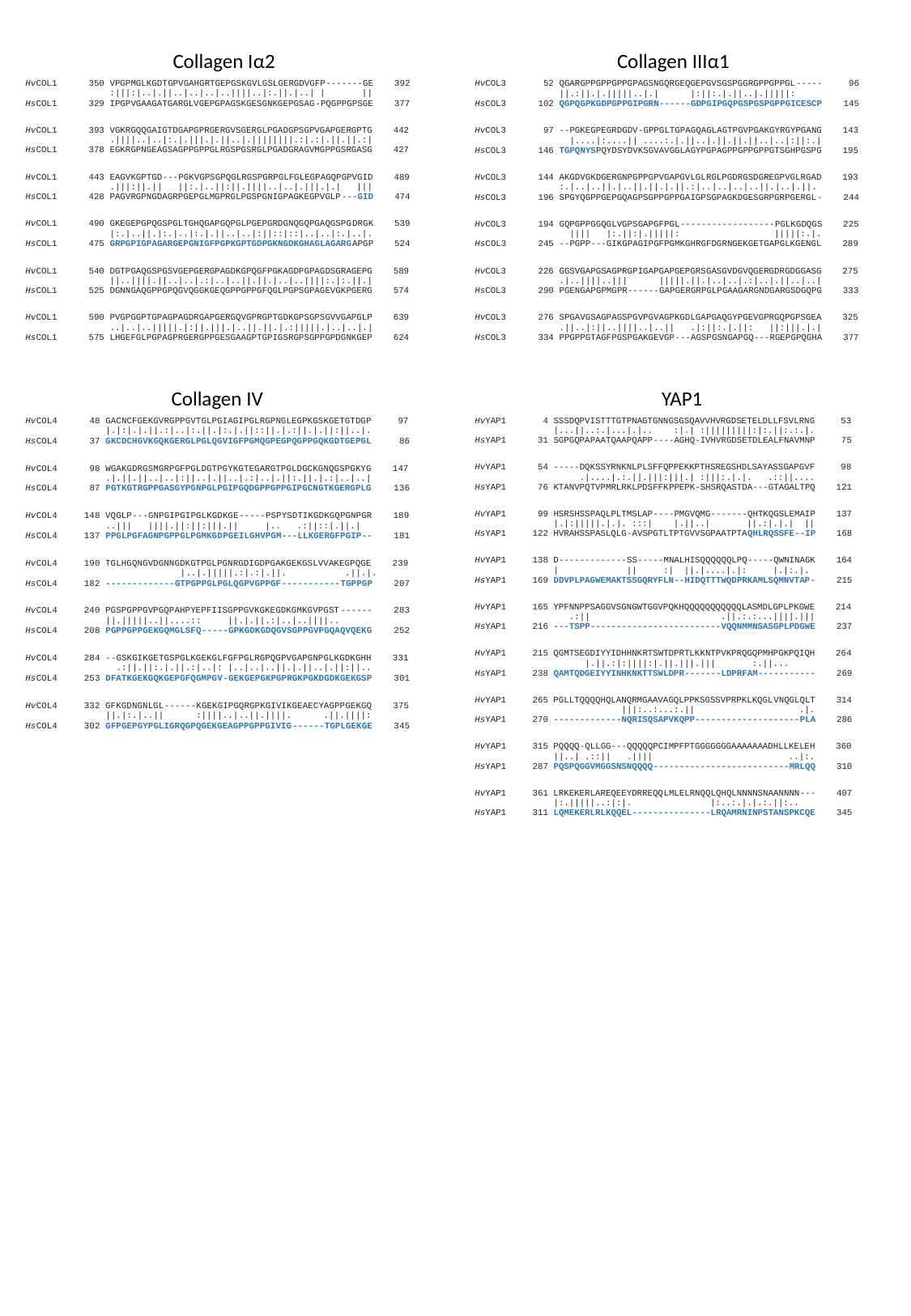

Collagen Iα2
Collagen IIIα1
Collagen IV
YAP1

Supplement: Supplementary file 1 — Supplementary file1 (PPTX 62 KB) [file 441_2024_3874_MOESM1_ESM.pptx]
