# Supplementary material for: Dynamic relationship among extracellular matrix and body wall cells in Hirudo verbana morphogenesis
Source: Cell Tissue Res. 2024 Mar 1;396(2):213–29. doi: 10.1007/s00441-024-03874-x (PMC11055932; doi:10.1007/s00441-024-03874-x)
Supplement: Supplementary file 2 — Supplementary file2 (DOCX 406 KB) [file 441_2024_3874_MOESM2_ESM.docx]

Animal model description

The body of an adult *Hirudo verbana* can reach a length of 7/8 cm in length and about 1 cm in diameter and shows a simple organization from an anatomically point of view. A muscular body sac contains the gut, the nervous system, blood vessels and other tissues that are embedded in a connective tissue characterized by widely spread fibroblasts and scarce collagen fibres (Sawyer 1986; de Eguileor et al. 2001; De Eguileor et al. 2004; Tettamanti et al. 2004; Grimaldi et al. 2006, 2010). The body wall is characterized by a cuticle, a monolayered epithelium and thick layers of muscle tissue, made of helical fibers. Muscle fibers are mononucleated and organized into groups of 10/15 elements completely separated from their neighbours by a scarce ECM. Each group is lined by a connective capsule and the groups of muscle fibers are further subdivided in sectors by connective tissue and by dorsoventral muscles, as visible in cross-sectioned body wall (Fig. S1).


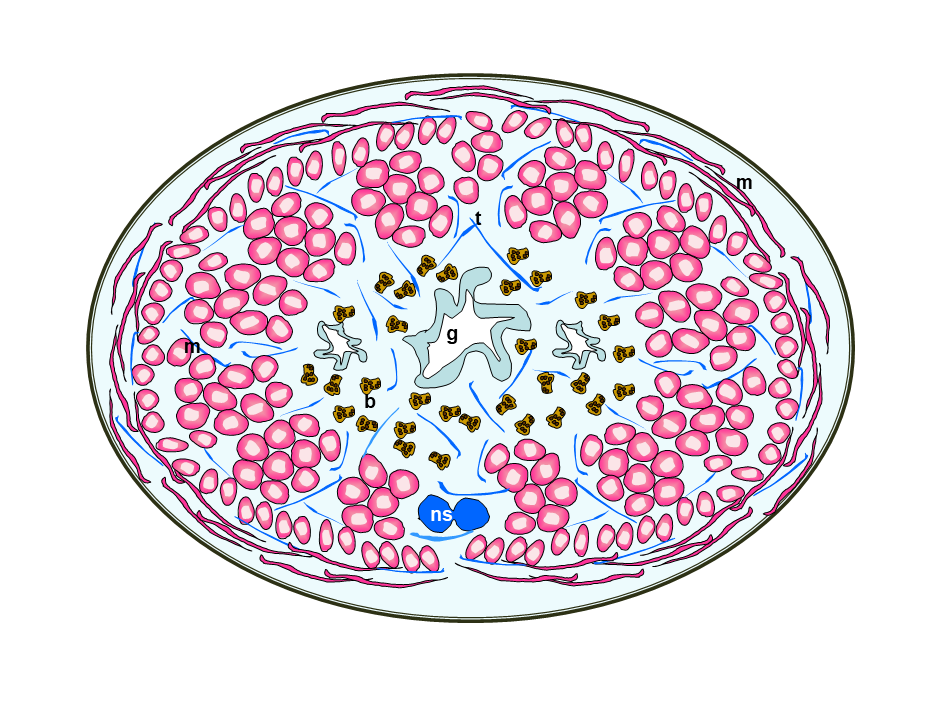


**Fig. S1**  Drawing representing the cross-sectioned body of *H.verbana*. Under the epithelium, muscle fibers (m) are oriented circularly, obliquely and longitudinally. Under the compact muscle wall, telocytes (t), botryoidal tissue (b), gut (g), with lateral diverticula, and the ventral nervous system (ns) are embedded in a loose connective tissue.

The loose connective tissue is populated by different types of resident cells, such as macrophages, granulocytes, NK cells, fibroblasts, that are represented in limited amounts, as well as telocytes (De Eguileor et al. 2000; de Eguileor et al. 2001; Grimaldi et al. 2004; Pulze et al. 2017). Leech fibroblasts are spindle-shaped cells with oval nucleus and a lipid droplets-filled cytoplasm, whereas telocytes are characterized by a small cellular body from which very long and thin cytoplasmic processes ramify. Their branches engage both -cell and cell-matrix contacts, thus forming a complex network interconnecting muscles, nervous system, blood vessels and collagen bundles (Pulze et al. 2017).
